# Editorial Comment to Rare case of immunoglobulin G4‐related disease arising in gonadal glands with long‐term remission without steroid treatment: Discussion and literature review

**DOI:** 10.1002/iju5.12288

**Published:** 2021-03-28

**Authors:** Yohei Sekino, Jun Teishima

**Affiliations:** ^1^ Department of Urology Graduate School of Biomedical and Health Sciences Hiroshima University Hiroshima Japan

Immunoglobulin G4‐related disease (IgG4‐RD) is a newly recognized and immune‐mediated fibrotic disease characterized by mass‐forming lesions with elevated serum IgG4 concentrations.^1^ A recent review reported that IgG4‐RD often manifests in multi‐organ systems: the biliary tree, salivary glands, periorbital tissues, kidneys, lungs, lymph nodes, meninges, aorta, breast, prostate, thyroid, pericardium, and skin.^2^ In this case report, Isobe *et al*. reported a rare case of IgG4‐RD in gonadal glands.^3^

In this case report, magnetic resonance imaging showed that the mass was found in the left epididymis and right seminal vesicle. Although testicle tumor markers and soluble interleukin‐2 were not elevated, the patient underwent orchidectomy because magnetic resonance imaging findings highly suspected seminoma or malignant lymphoma. After the diagnosis of IgG4‐RD, high levels of serum IgG4 was observed. These findings indicate that clinicians should consider the possibility of IgG4‐RD and check the level of serum IgG4 when examining patients with the atypical clinical course.

In this case report, the mass in the right seminal vesicle was reduced after left orchidectomy without further treatment. Based on the International Consensus Guidance Statement on the Management and Treatment of IgG4‐RD, “watchful waiting” may be appropriate in patients with no symptoms.^4^ Indeed, a recent review reported that 52/235 (22%) patients were not treated because of no symptoms, diabetes mellitus, history of malignant disease, spontaneous regression, and refusal to undergo treatment.^5^ Further studies are needed to determine the appropriate treatment of IgG4‐RD.

## Conflict of interest

The authors declare no conflict of interest.
